# Workability and Muscle Strength in Patients With Seropositive Rheumatoid Arthritis: Survey Study Protocol

**DOI:** 10.2196/resprot.6449

**Published:** 2017-03-02

**Authors:** Carolin Berner, Ludwig Erlacher, Michael Quittan, Karl Heinrich Fenzl, Thomas Ernst Dorner

**Affiliations:** ^1^ Kaiser Franz Josef Hospital, Sozialmedizinisches Zentrum-Süd Department of Rheumatology and Osteology Vienna Austria; ^2^ Karl Landsteiner Institute for Autoimmune Diseases and Rheumatology Vienna Austria; ^3^ Kaiser Franz Josef Hospital, Sozialmedizinisches Zentrum-Süd Department of Physical Medicine and Rehabilitation Vienna Austria; ^4^ Karl Landsteiner Institute for Remobilisation and Functional Health Vienna Austria; ^5^ Institute of Social Medicine Centre for Public Health Medical University of Vienna Vienna Austria

**Keywords:** rheumatoid arthritis, work ability, frailty, muscle strength, functional disability, lower extremity function, sexual functioning

## Abstract

**Background:**

Rheumatoid arthritis (RA) and other rheumatic conditions not only fundamentally affect patients’ quality of life and physiological needs but are also negatively associated with work ability. The costs of poor work ability, which, in sum, are more than treatment costs, pose an economic burden to society and patients. Work ability in RA appears to be multifactorial; symptoms such as pain, swelling, and stiffness play a major role, as these directly affect functional disability. Also, RA patients typically suffer from reduced muscle strength. Lower extremity function and grip strengths especially impair their quality of life. However, the role of muscle strength and disease activity as determinants of work ability have not yet been studied.

**Objective:**

The primary objective of this study is to compare work ability in working-age participants with seropositive RA and with high and low disease activity; the secondary objective is to evaluate the association of muscle strength, functional ability, and frailty with work ability.

**Methods:**

This monocentric cross-sectional study will be conducted at a rheumatologic outpatient clinic and day hospital with approximately 100 seropositive RA patients aged <65 years. A clinical disease activity index as a measure for rheumatoid disease activity will be assessed during the patients’ routine visits at the clinic. Work ability, frailty, and functional disability will be evaluated with (self-reported) questionnaires as well as with physical tests (Work Ability Index/Score; Health Assessment Questionnaire Disability Index; Survey of Health, Ageing, and Retirement in Europe Frailty Instrument; Short Physical Performance Battery). Muscle strength will be determined with dynamometer measurements of isometric hand grip strength and quadriceps femoris muscle contraction strength. Sleep quality (Medical Outcomes Study Sleep Scale) and sexual functioning as physiological needs will additionally be determined with self-reported questionnaires.

**Results:**

For this study funding has already been awarded and enrollment has been completed. Data are currently being evaluated.

**Conclusions:**

This study will evaluate the association of work ability with modifiable parameters such as muscle strength and functional ability. It will provide further insights into work ability in RA and its associated risk factors. Any evidence of association will motivate further research, and the findings might encourage interventions focused specifically on improving muscle strength and lower extremity function to positively affect work ability.

**Trial Registration:**

ClinicalTrials.gov (NCT02581852); https://clinicaltrials.gov/ct2/show/NCT02581852 (Archived by WebCite at http://www.webcitation.org/6oNcelHtQ)

## Introduction

### Background

Rheumatoid arthritis (RA) is a chronic, systemic autoimmune disease that is most prevalent in individuals aged 40 years and above. RA affects about 1% of the world population, and the risk of developing RA is markedly higher in women. This disease is the most common form of chronic joint inflammation and causes joint pain, swelling, reduced muscle strength, and, as a consequence, impaired physical function [1]. In our experience, due to physical limitations, RA patients often suffer from impairment in their social life and their work life. Working is an important predictor of physical health–related quality of life in arthritis patients [2], and reduced muscle strength can cause physical limitations and a more frail condition, which in turn might have an impact on work ability. Beside obvious symptoms that directly cause physical limitations, physicians less often pay attention to physiological needs such as sleep and sexual functioning, which are also negatively associated with RA and in turn might unfavorably impact work ability.

### Rheumatoid Arthritis and Work Ability

Chronic diseases such as RA are negatively associated with work ability. Work ability, as described by Ilmarinen from the Finnish Institute of Occupational Health, is the interaction of individual determinants (health, competence, and attitudes) and the work environment [3]. It is determined by an individual’s perception of the demands at work and the ability to cope with them [4]. Although RA-induced work disability rates seem to decrease because of new therapeutic concepts, RA is still a fundamental burden for many patients [5]. The costs of work disability in rheumatic conditions are high, generally higher than the treatment costs [6]. Furthermore, from the patients’ perspective, work disability significantly affects their basic income [7].

Work disability in RA appears to be multifactorial; symptoms such as pain, swelling, and stiffness play a major role, as they directly affect functional disability [8]. RA is associated with loss of muscle mass and diminished strength. In healthy individuals, only a weak association between muscle strength and workability could be found [9]. The influence of strength on the work ability of RA patients has yet to be determined.

### Rheumatoid Arthritis and Muscle Strength

Body composition, particularly the amount of lean mass in the arms and legs, is associated with disability in RA patients. A large number of RA patients suffer from an increased loss of muscle mass with a significant impact on these patients’ quality of life [10]. This condition is commonly known as rheumatoid cachexia and has been reported in two-thirds of all RA patients, including patients with stable RA [11,12]. In geriatric patients, cachexia is associated with higher-than-normal concentrations of tumor necrosis factor-α (TNF-α), interleukin (IL)-1, and IL-6; a reduction in these proinflammatory cytokines is associated with weight gain [12,13]. The loss of body cell mass in RA patients is associated with proinflammatory cytokine–induced altered energy metabolism and intake despite a theoretically adequate diet [14,15].

In an x-ray computed tomography–based study, RA patients had a significantly higher body mass index and fat area but lower muscle area, muscle density, and muscle strength than healthy individuals. Furthermore, a higher degree of joint destruction and disease activity were shown to be associated with higher muscle deficits and impaired muscle strength [16]. Stucki et al [17] found that in patients with RA quadriceps muscle strength explained 12% of the variance in the self-reported activities of daily life, and women with RA were reported to have 20% lower quadriceps strength than the controls [18]. Isotonic and isometric hand exercise in RA patients can decrease pain and disease activity and increase muscle strength and function as well as quality of life [19]. Hand grip strength was shown to discriminate between various disease states of RA and seems to return to near-normative level when the disease is in remission [20].

### Rheumatoid Arthritis and Sleep

Most patients with RA experience insomnia, general fatigue, and mental fatigue, which negatively affect their physical and cognitive functioning and health. Arthritis pain can lead to sleep deprivation and a lack of sleep, which, in turn, contribute to increased pain and fatigue [21-24]. Poor sleep has also been shown to negatively influence work ability in otherwise healthy individuals [23].

### Rheumatoid Arthritis and Sexual Functioning

All aspects of life may be affected by RA. Pain, stiffness, and fatigue may impair not only functional disability but also sexual functioning in RA patients. As summarized by a recent review, aside from functional problems, depression, anxiety, a negative body image, reduced libido, and the application of certain drugs can negatively influence sexual activities [25].

### Rheumatoid Arthritis and Frailty

Frailty, as defined by Fried et al, refers to a state of increased vulnerability to external and internal stressors caused by a reduction in physiological reserves. It has been described as a clinical phenotype of unintentional weight loss, low energy, slow walking speed, low physical activity, and low grip strength (weakness). The presence of 3 out of 5 of these criteria indicates frailty, and the presence of 1 or 2 indicates a prefrailty state [26-28]. Frailty has been associated with a higher risk of adverse health outcomes, mortality, hospitalization, and functional impairment [26,29,30]. Several studies have shown a heightened inflammatory state in frail adults [31], and similar to other markers of frailty, gait speed has been shown to be associated with elevated levels of inflammation markers such as C-reactive protein (CRP), IL-6, and TNF-α [32].

The population of elderly and frail individuals with RA is increasing [24]. However, although older people are more likely to develop long-term illnesses, muscle loss, and reduced strength, age is not the sole predictor of frailty, and it may occur in younger patients [33]. Generally, RA patients appear to be more prone to frailty because loss of muscle mass and an increase in proinflammatory cytokines are clinical characteristics that are also present in young RA patients. The association of the frailty of young RA patients and work ability has yet to be examined.

### Aims and Objectives

The primary objective is to assess work ability in patients with seropositive RA in the working age and the association of work ability with disease activity (high, medium, low, or remission). The secondary objectives are to assess the association of muscle strength, lower extremity function, functional ability, and frailty with work ability in RA and determine the association of disease activity with selected physiological needs (sleep quality, sexual functioning) in RA.

## Methods

### Study Design

This study will be conducted as a monocentric cross-sectional study in seropositive RA patients at the rheumatology outpatient clinic and ambulatory day clinic of the Second Medical Division of the Sozialmedizinisches Zentrum (SMZ)-Süd, Vienna, Austria. Eligible patients will be consecutively included during an expected period of 1 year. Ethical review committee approval was obtained from Gemeinde-Wien (EK 15-173-0915). The study is registered at ClinicalTrials.gov [NCT02581852].

### Study Population

The study will involve 100 patients of working age between 18 and 65 years with seropositive RA according to 2010 European League Against Rheumatism classification. Eligible patients will be identified by staff members, and eligibility will be confirmed by the physician in charge at the outpatient or day clinic. Written informed consent will be obtained from each patient before enrollment. Patients who do not wish to sign the informed consent, are unable to follow advice for physical measurements and understand interview questions, and those with severe comorbidities will be excluded from the study. Questionnaires will be provided in German, English, Turkish, and Serbo-Croatian. Translations will be provided by Frauengesundheitszentren-Integration and Health Center (SMZ-Süd).

### Sample Size

The sample size was calculated to determine the difference in work ability between patients with high versus low disease activity. We estimate the percentage of patients with good work ability to be 40% in patients with high disease activity and 80% in patients with low disease activity. Furthermore, we estimate that 70% of the included participants will have a low disease activity. With an alpha risk of .05 and a beta risk of .2 accepted in a 2-sided test, 71 patients need to be included (results were obtained with nQuery Advisor 7.0, Statcon). With a 30% rate of loss assumed (because of noncompliance during physical tests, lack of understanding, or refusal to participate), a total of 100 included patients will be required.

### Data Collection and Procedures

Demographic, clinical data, and disease activity will be assessed through clinical examination and administration of an interview questionnaire. The demographic characteristics include gender, age, marital status (married/common law, single/widowed/ divorced), highest level of education (compulsory schooling, secondary school graduation, higher education), and type of occupation.

Disease-specific clinical characteristics include disease duration (months) and current medication use for each of the following categories: analgesics/nonsteroidal anti-inflammatory drugs, disease-modifying antirheumatic drugs, biologic agents (biologicals), and drugs for other medical conditions.

Pain will be assessed via a visual analog scale (VAS), a unidimensional measure of pain intensity that has been widely used in diverse adult populations, including those with rheumatic diseases. The VAS consists of a 10 cm line anchored by verbal descriptors (1=no pain at all, 10=maximum pain) [34]. The patient will be asked to place a line perpendicular to the VAS line to indicate his or her current pain intensity.

Overall disease activity will be measured with the Clinical Disease Activity Index (CDAI). The CDAI is validated and widely used [35], with the scoring done as per the following formula: CDAI = SJC-28 + TJC-28 + PGA + EGA, where SJC-28 is the Swollen 28-Joint Count (shoulders, elbows, wrists, metacarpophalangeal joints, proximal interphalangeal joints including thumb interphalangeal joint, knees), TJC-28 is the Tender 28-Joint Count, PGA is the Patient Global Assessment (patient’s self-assessment of the overall RA disease activity on a scale of 1 to 10, where 10 is the maximal activity), and EGA is the Evaluator’s Global Assessment (evaluator’s assessment of the overall RA disease activity). As proposed by the American College of Rheumatology 2008, the CDAI score will be interpreted as follows: remission = CDAI ≤ 2.8, low disease activity = CDAI > 2.8 and ≤ 10, moderate disease activity = CDAI > 10 and ≤ 22, and high disease activity = CDAI > 22.

Laboratory assessments include CRP (mg/dL), IL-6 (pg/mL), and TNF-α (ng/mL). For the detection of IL-6 and TNF-α, high-sensitivity enzyme-linked immunosorbent assay will be used, assayed in duplicates. The CRP will be measured with the patients’ routine laboratory assessments. Blood samples will be analyzed at the Institute for Laboratory Medicine of the SMZ-Süd, Vienna, Austria.

### Primary Outcome

The primary outcome is self-reported work ability measured by the Work Ability Index (WAI), the most commonly used instrument to assess work ability, with an adequate test-retest reliability [36]. The WAI is a questionnaire consisting of 7 subscales: (1) current work ability compared with the lifetime best, (2) work ability in relation to the demands of the job, (3) number of current diseases diagnosed by a physician, (4) estimated work impairment because of disease, (5) sick leave during the past 12 months, (6) own prognosis of work ability 2 years from now, and (7) mental resources. The cumulative index of WAI ranges from 7 to 49 points, divided into the following 4 categories: poor (7-27 points), moderate (28-36 points), good (37-43 points), and excellent work ability (44-49 points) [37-39].

The methodological problem with using the WAI in unemployed patients is that most points of the self-evaluation reference to the current work setting. Thus, for unemployed and early retired patients, only 1 dimension of WAI, the Work Ability Score (WAS), will be assessed. The WAS comprises only the first WAI question: work ability compared with the lifetime best.

Justification on the use of WAS as 1 single question is based on previous studies that showed a high correlation between WAI and WAS [40,41]. As proposed by Gould et al [42], the classification of WAS will be conducted according to WAI as follows: poor (0-5 points), moderate (6-7 points), good (8-9 points), and excellent work ability (10 points).

### Secondary Outcomes

#### Muscular Strength Measurement

Muscle strength measurement will be performed by a trained sport scientist at the Institute of Physical Medicine situated adjacent to the rheumatologic clinic.

##### Musculus Quadriceps Femoris Maximum Voluntary Contraction Strength

Quadriceps muscle strength will be measured with an isokinetic dynamometer. The patients will sit straight with 90° flexion in the hips and with fixed hip and thigh support and arms crossed. The ankles will be fixed in a flexed position to the dynamometer, and a measuring box (Chatillon, Ametek Inc) is connected to the ankle via a length-adjustable rope. The patient will be instructed to perform 1 maximal voluntary contraction of the quadriceps muscle. Strength will be assessed 3 times for both legs with a 2-minute break between measurements. The mean value of both legs will be used in the statistical analysis.

##### Maximal Voluntary Isometric Hand Grip Strength

The maximum grip strength will be measured with a portable Jamar hydraulic hand dynamometer (Patterson Medical). The patients will be examined in a standard position, in which they sit upright with their upper arm adducted and the elbow flexed at 90°. The dynamometer will be used according to the instructions in the operating manual. The dynamometer will be placed in a patient’s hand, and after the instruction is given, 3 maximum voluntary grip strength contractions will be performed with each hand. Measurements will be done in an alternating order with a 2-minute break between measurements. The mean value of each hand will be recorded for analysis.

#### Functional Disability

The Health Assessment Questionnaire Disability Index (HAQ-DI) will be used to assess the extent of patients´ self-reported functional disability. The HAQ-DI has been used to measure functional status in RA in multiple settings and languages since 1980, and it has shown good reliability and validity [43]. It examines patients’ usual abilities in using their regular equipment during the past week. The scoring of the HAQ-DI is patterned after the functional classes of the American Rheumatism Association/American College of Rheumatology. A total of 20 questions are included in the following 8 categories of functioning: dressing, rising, eating, walking, hygiene, reach, grip, and usual activities.

Self-reported difficulties to perform these activities are scored on a scale from 0 to 3, representing normal (no difficulty) (0), some difficulty (1), much difficulty (2), and unable to do (3). For any component question, the highest score determines the score for that respective domain. The overall disability index is a value between 0 (no functional disability) and 3 (severe functional disability), representing the average score across the domains [44].

#### Survey of Health, Ageing, and Retirement in Europe Frailty Instrument

Frailty will be assessed with the Survey of Health, Ageing, and Retirement in Europe Frailty Instrument (SHARE-FI). This instrument was developed on the basis of the results of the Survey of Health, Ageing and Retirement in Europe and serves as a rapid measurement tool with good predictive validity. The assessment comprises 5 variables: grip strength measurement and 4 questions related to weakness, exhaustion, slowness, and activity level. With the use of the 5 variables, DFactor scores will be calculated with the gender-specific SHARE-FI formula. For each participant, the frailty score will be computed, and the participants will then be categorized as nonfrail, prefrail, or frail [45].

#### Short Physical Performance Battery

Lower extremity function will be measured with the Short Physical Performance Battery (SPPB). The SPPB is a group of measures including gait speed, chair stand, and balance tests. For each test, a 5-level categorical score will be assessed, with 0 representing inability to complete the test and 4 representing the highest level of performance. The summary score ranges from 0 (worst performance) to 12 (best performance). The SPPB has been shown to have good predictive validity, has been used in the RA population before, and can be used as a predictive tool for possible disability and mortality in older people [46-49].

#### Medical Outcomes Study Sleep Scale

The patients’ quality of sleep will be assessed with the Medical Outcomes Study Sleep Scale (MOS-SS) questionnaire, which was created as a part of the Medical Outcomes Study, a large public health initiative that also developed practical tools for the routine monitoring of patient outcomes [50].

The MOS-SS is a 12-item self-report questionnaire involving a retrospective assessment over the past 4 weeks. The MOS-SS measures 6 sleep dimensions: (1) initiation (time to fall asleep in minutes), (2) quantity (hours of sleep each night), (3) maintenance, (4) respiratory problems, (5) perceived adequacy, and (6) somnolence. The last 4 items will be assessed via a 6-item scale ranging from “all the time” to “none of the time” [51].

The questionnaire yields 2 sleep problem indexes and 6 scores, of which sleep problem indexes I and II and the sleep disturbance scale were shown to have acceptable validity in RA patients [52]. Furthermore, the MOS-SS has been applied in several arthritis studies, and its good reliability and validity have been established [53]. Additional questions will assess the significance of pain in sleeping difficulties and the frequency of pain and sleep medication use.

#### Sexual Functioning

Sexual functioning will be measured via a self-assessment questionnaire individually designed for this study. Standardized questionnaires on sexual functioning such as the questionnaire for screening sexual dysfunctions or gender-focused questionnaires such as the Female Sexual Function Index are extensive and very detailed. For questions related to sexuality, people tend to react apprehensively, so data collection (completion of the questionnaires) will be conducted in the rheumatology outpatient department (not as an online or mail survey). A short screening tool that focuses on the main problems that RA patients face with regard to sexual function was created.

Based on a recent review [25] and a multicenter study [54] on the impact of RA on sexual function, the questionnaire was designed consisting of 2 sections addressing sexual disability (difficulties in performing sexual intercourse), represented by question 1, and sexual drive (reflected in sexual desire and satisfaction), represented by questions 2 to 5. Scoring ranges from 1 to 10, and anchor points are set according to the question. The overall sexual functioning score ranges from 5 points (poor sexual functioning) to a maximum of 50 points.

### Statistical Analysis

All statistical computations will be performed with SPSS version 22.0.2 (IBM Corp). The primary hypothesis of the study, “A difference in work ability and muscle strength exists in patients with high versus low disease activity,” will be tested with a Pearson chi-square test. For the descriptive statistics, mean values will be calculated for continuous variables, and categorical variables will be presented in percentages. Continuous parameters will be checked for normal distribution (Kolmogorov-Smirnov test, Levene test, histogram, and Q-Q plot check), and an unpaired Student *t* test will be performed, as appropriate. Otherwise, the Mann-Whitney U test will be used to identify differences between patients with high versus low disease activity. For categorical parameters, a Pearson chi-square test or Fisher exact test will be used if the cell count is <5. Binary logistic regression will be used to evaluate the impact of frailty, functional ability, and muscle strength on work ability. For metric data, Pearson correlation or Spearman rank correlation (given outliers or nonlinear but monotone associations) will be used to evaluate associations of interest. A *P* value of <.05 will be considered statistically significant for all tests.

## Results

For this study, funding has already been awarded and enrollment has been completed. Data are currently being evaluated.

## Discussion

### Summary

This cross-sectional study was designed to evaluate work ability in a population of seropositive RA patients and its association with RA disease activity, muscle strength functional disability, lower extremity function, and frailty. This study will also assess the quality of sleep and sexual functioning in RA and the respective association of these factors with disease activity.

### Implications for Research and Clinical Practice

On the basis of the large number of people affected, RA not only lowers patients’ quality of life but also poses an economic burden to both patients and society in general [55]. The direct health care–related costs of RA are dominated by in-patient care, but the total costs are found to be mainly related to work disability and temporary or permanent loss of work [55,56].

This study will allow the estimation of the rates of RA patients at risk for incapacity for work and will evaluate the association of work disability with modifiable parameters such as muscle strength and functional ability, which has not been investigated in RA before. It will provide further insights into work ability in RA and its associated risk factors. Any evidence of association may motivate further research, and the findings might encourage interventions focused specifically on improving muscle strength and lower extremity function to positively affect work ability. The outcomes of this study may also motivate clinicians to screen for work ability and modifiable parameters in the RA population.

Aside from work ability, quality of sleep and good sexual functioning beneficially affect one’s quality of life [57]. This study will provide insights into the rates of RA patients with impaired sleep and sexual dysfunction and the association of these functions with RA disease activity. The findings may encourage future research and interventions for the improvement of RA-associated problems, which tend to be poorly addressed in routine care. A screening tool for sexual functioning in RA patients was used in this study, and the applicability of the tool will be evaluated.

### Strengths and Limitations

The study is limited by its cross-sectional design, which allows us to draw a conclusion about association but not causality. However, as we intend to analyze multiple outcomes and potential risk factors, we considered this approach to be ideal for generating hypotheses for further interventional approaches or longitudinal studies. Another potential limitation might be the pain-related noncompliance of patients during physical measurements. High disease activity and pain may possibly lead to some degree of bias in the results.

An advantage of this study is the availability of patients from various settings. Through recruitment at an outpatient clinic and a day clinic, the whole spectrum of different disease activities can be investigated. All study-related measures can be performed in short time periods without the necessity of a second appointment, thereby assuring high patient compliance and a marginally low drop-out rate.

## Abbreviations

CDAI: Clinical Disease Activity Index
CRP: C-reactive protein
EGA: Evaluator’s Global Assessment
HAQ-DI: Health Assessment Questionnaire Disability Index
IL: interleukin
MOS-SS: Medical Outcomes Study Sleep Scale
PGA: Patient Global Assessment
RA: rheumatoid arthritis
SHARE-FI: Survey of Health, Ageing and Retirement in Europe–Frailty Instrument
SJC-28: Swollen 28-Joint Count
SMZ: Sozialmedizinisches Zentrum
SPPB: Short Physical Performance Battery
TJC-28: Tender 28-Joint Count
TNF-α: tumor necrosis factor-α
VAS: visual analog scale
WAI: Work Ability Index
WAS: Work Ability Score
